# Measurement and Assessment of Head-to-Helmet Contact Forces

**DOI:** 10.1007/s10439-025-03677-3

**Published:** 2025-01-25

**Authors:** Turner Jennings, Aidan Tillman, D’mitra Mukasa, Michael Marchev, Sinan Müftü, Rouzbeh Amini

**Affiliations:** 1 Department of Mechanical and Industrial Engineering, Northeastern University, 360 Huntington Avenue, Boston, MA 02115 USA; 2 Department of Bioengineering, Northeastern University, 360 Huntington Avenue, Boston, MA 02115 USA

**Keywords:** Helmet fit, Ergonomics, Sensors, Sex differences, Head shape, Kevlar composite, Homework

## Abstract

**Purpose:**

To evaluate the population variation in head-to-helmet contact forces in helmet users.

**Methods:**

Four different size Kevlar composite helmets were instrumented with contact pressure sensors and chinstrap tension meters. A total number of 89 volunteers (25 female and 64 male volunteers) participated in the study. The length, width, and circumference of their heads were measured and each volunteer was assigned a helmet size. Volunteers were asked to wear the helmet in three different configurations and the chinstrap tension and contact force between the head and each of the seven interior pads were recorded.

**Results:**

The majority of forces measured on any individual pad were between 0 and 5 N. However, some users exhibited pressure points with forces as high as 30 N. The contact force distribution is non-uniform across the interior of the helmet, with the largest force concentrated at the front. Head shape is a major driver of the observed contact force. There was a statistically significant difference between female and male volunteers, and between groups with different experience levels.

**Conclusions:**

The fit of helmet systems is highly subject specific. The current metrics used to assign helmet sizes may not accurately predict correct helmet fit.

**Supplementary Information:**

The online version contains supplementary material available at 10.1007/s10439-025-03677-3.

## Introduction

Traumatic brain injury (TBI) due to sports collisions and road traffic incidents represents a significant public health burden [1]. It has been rigorously shown in previous research that the use of helmets can alleviate this burden by reducing incidence of serious brain injuries [2, 3]. As a result, research in improving helmet systems has become a high priority across a variety of helmet use cases [4–6]. While the majority of the recent research has focused on materials development, the ergonomics of the helmet system is a critical and understudied factor to consider in evaluating approaches to increase helmet effectiveness.

The role of helmet fit in brain injury has been comprehensively established in numerous fields of helmet development. Researchers have previously reported higher incidence rate and severity of concussions in individuals with a poor helmet fit while cycling [7–10], riding motorcycles [11], playing ice hockey [12, 13], lacrosse [14], American football [15–17], and skiing or snowboarding [18]. In such studies, helmet fit was evaluated by qualitative measures, either self-reported by the helmet user or evaluated by a trainer or study facilitator. As a result, while providing strong evidence of a relationship between helmet fit and head injury risk, a comprehensive criterion to describe helmet fit is not explored therein.

Other researchers have quantitatively described the relationship between helmet fit and head injury for a subset of the applications discussed above. Chang et al. investigated the role of helmet fit in injury risk during motorcycle crashes [19]. They found that a larger contact area between the head and helmet pads led to the impact forces being more easily transferred onto the head from an outside impact. As a result, a helmet that fits tighter would tend to carry a higher risk of brain injury. Their results were corroborated by Mills et al., who found that a higher head/helmet friction coefficient led to larger linear and angular accelerations of the head [20]. Similarly, Bonin et al. found through drop tower experiments that use of an oversized motorcycle helmet tended to lead to lower peak accelerations in the head [21]. However, an oversize helmet also increases the risk of helmet ejection during an impact event in an uncontrolled environment. Since such an ejection significantly increases the risk of injury [22], a direct relationship between helmet size and injury risk cannot be conclusively drawn from controlled drop tower experiments alone.

The studies discussed above strongly suggest that there is a negative correlation between the quality of helmet fit and the risk of head injury. However, the criteria for a well-fitted helmet remain ill-defined. Previous literature has described measures based on the geometry of the head and helmet or on qualitative measures of “snugness" [23–25]. However, a lack of quantitative fitting data beyond helmet size and shape prevents the development of more precise criteria. Other researchers have measured the fit conditions between the head and helmet using forces rather than the system geometry. Yu et al. measured the fit force between a bicycle helmet and a Hybrid III surrogate headform using load cells arrayed across the surface of the head form [26]. They found a total contact force between the head and helmet of between 0.8 and 2.2 Newtons, with the majority of the load concentrated on the top of the head. Notably, Cobb et al. and Liu et al. have both measured significant dimensional differences between the Hybrid III headform and population average anthropometry which may limit the applicability of fit forces measured on headforms [27, 28].

While previous researchers have measured the helmet-to-head forces on headforms, limited data exist quantitatively measuring helmet fit metrics using human subject data. Jadischke used a skullcap instrumented with contact pressure sensors to measure the force distribution across the inside of American football helmets and assess the variation with different helmet users [29]. Their data showed a significant variance in contact force magnitude at different points on the head between human subjects, highlighting the role of head shape in determining head/helmet fit characteristics.

Researchers previously examining helmet fit have largely focused on helmets for sports or automotive safety rather than for protection against higher velocity threats, as seen in military or law enforcement helmets. To date, the role of fitting in military or law enforcement helmets has mostly been evaluated from the perspective of user-comfort [30, 31] and the risk of neck injury [32, 33]. Kuo et al. performed an analysis of fitment in composite helmet systems by measuring the standoff distance and contact pressure [34]. Their results suggest that thicker helmet padding results in higher forces between the head and the helmet. In their study, they used only a size large helmet and tested twenty male volunteers, so details of how the fit relationship may change with helmet size or population variation remains unclear.

Samil and David performed an analysis of helmet fit through a qualitative questionnaire and by measuring the head/helmet contact force, while volunteers were jogging on a treadmill [35]. The results of their questionnaire indicate that the majority of participants experienced discomfort while wearing the helmets. Additionally, their study showed that the mean and maximum pressures observed across the inside of the helmet vary from user to user between 0–6 kPa and 0–10 kPa, respectively. However, they did not analyze the spatial distribution of pressures, leaving the relationship between maximum pressures and the areas of user discomfort unknown.

Existing studies of helmet ergonomics for military or law enforcement applications have not captured sufficient quantitative data to describe the spatial and population distribution of helmet fit. Such a description, incorporating both spatial and physical characteristics, is critical toward establishing an “appropriate fit” criterion for such helmet systems to reduce the risk of injury. In the study presented herein, we have expanded on the framework established in previous studies [29, 34] and performed a comprehensive measurement of the contact force between the head and helmet padding in a Kevlar composite helmet system. We have evaluated how the contact force distributions change with biological sex, preferred chinstrap tightness and experience level. We also investigated how the size and shape of the head and helmet affect the tightness at the head/padding interface. Thus, this study provides comprehensive statistical distributions of how fit of Kevlar composite helmet systems varies from person to person, with experience level and biological sex.

## Methods

**Fig. 1:**
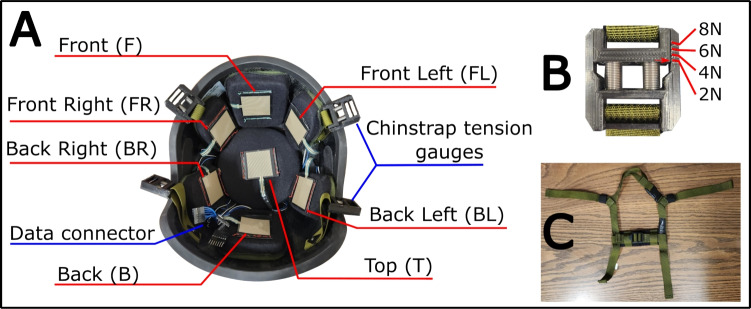
Measurement system diagram. **A**: Helmet with force sensitive resistor notation marked in red, other key features marked in blue. **B**: Detail of the chinstrap tension gauges. **C**: Detail of the helmet chinstrap system, aligned to correspond to the helmet shown in **A**

Contact force testing was performed using an open market Kevlar composite helmet system similar to those used by law enforcement. Four helmets were purchased in sizes small, medium, large, and extra large (Ace Link Industrial, Inc., Irvine, CA; Security Pro USA, Los Angeles, CA). Each helmet shell was incorporated with an aftermarket foam helmet liner system (Oregon Aero, Inc., Scappoose, OR) designed for this helmet geometry to provide a consistent basis for comparison. The liner system consisted of seven pads: two trapezoidal pads which were installed at the front and rear of the helmet, one circular pad installed in the crown, and four oval pads installed at the sides of the head in the front and back, as shown in Fig. 1.

A force sensitive resistor (FSR) (Adafruit Industries, LLC. New York, NY) was installed on each pad. To mount each FSR, a flexible rim was created around the perimeter of the sensor using electrical tape. The rim was sewn onto the cloth exterior of the padding, which allowed the sensors to flex and conform to the shape of the pad. Measurements were recorded using an Arduino Mega microcontroller. Because flexure in the FSR was observed to change the measured force value, the pressure measurement on the surface of the pad was calibrated with the FSR and pad installed in the helmet. FSRs calibration curves were explicitly measured after the sensors were installed in the helmet. Additional details of the calibration procedure, as well as the derived curves, can be found in the supplemental material.

The helmet system uses a chinstrap suspension system which connects to the helmet shell at four points. To measure the tension in the chinstrap, a custom mechanical force gauge was developed which replaced the original adjustment buckles in the chinstrap. The force gauges enabled measurement of the chinstrap tension via stretching of a spring in the buckle, with a 2 N force resolution and a measurement range of 0–10 N. The force gauges developed were installed at all four connection points between the helmet and shell, as shown in Fig. 1.

The user study was performed with official exemption from the Northeastern University Institutional Review Board (IRB#23-03-15). A total of 89 volunteers with varying levels of experience participated in the study. Volunteers were facilitated from groups familiar with the use of similar helmet systems: local army reserve units, local Reserve Officers’ Training Corps (ROTC) battalions, and the Northeastern University Student Veterans Association. Additional volunteers from Northeastern University with no prior experience using the helmet systems were also facilitated. A summary of volunteer age and sex distribution is shown in Table 1 Participation in the study was anonymous, with only the volunteers age and sex being recorded. Of the volunteers, 25 self-identified as female and 64 self-identified as male. The average age of volunteers was 29±9 years.

**Table 1:** Volunteer distribution by sex and prior experience using the helmet system

	Female	Male	Total
No prior experience	9	9	18
ROTC cadet	9	14	23
Active duty/reserve	6	31	37
Veteran	1	10	11
Total	25	64	89

The study was facilitated by asking volunteers for their age, biological sex, and level of experience using the helmet system being evaluated. Level of experience was stratified into four factors: None (first time wearing the type of helmet used), Cadet (trained on equipment but minimal use), Active/reserve (trained on equipment and regular use), and Veteran (trained on equipment but no recent use). The length, width, and circumference of the participants head were then measured and recorded using a tape measure and layout calipers. The head length was measured front-to-back from the glabella to the furthest point at the back of the head. The head width was measured at the widest point of the skull above the ears. The head circumference was measured across the brow ridge, above the ears, and through the widest point on the back of the head. The correct size helmet was selected for the volunteer based on their measurements and the criteria outlined in the helmet operator’s manual [36].

Volunteers were instructed to put on the measurement helmet in three sequential configurations. First, the volunteer was instructed to put the helmet on and leave the chinstrap unbuckled (hereby referred to as “no-chinstrap" configuration). Next, the volunteer was instructed to fasten the chinstrap and tighten to their preferred comfort level (“user-comfort" configuration). Finally, the study facilitator adjusted the helmet chinstrap for the volunteer so that there was a uniform tension on the two front chinstrap buckles (“fixed-tension" configuration). A tension value of 4 N was selected to provide a uniform basis for comparison of contact forces with the chinstrap on but agnostic of user preference for chinstrap tightness. In all three cases, the contact force was measured on each of the seven interior pads. For the user-comfort configuration, the preferred chinstrap tension set by the volunteer was also recorded. Data were collected from the Northeastern University volunteers with no prior composite helmet experience prior to the implementation of the chinstrap tension gauges, so tension data and the fixed-tension configuration were not measured for that group.

It was found that the nape cushion shown in Fig. 1C caused medial compression of the left and right rear straps in the chinstrap system. As a result, the two rear chinstrap tension gauges carried a negligible load so the forces measured on the two rear gauges were ignored for all volunteers. Additionally, there were three instances of the chinstrap tension gauge breaking during volunteer testing. Mean imputation was used to fill in the missing values.

## Results

**Fig. 2:**
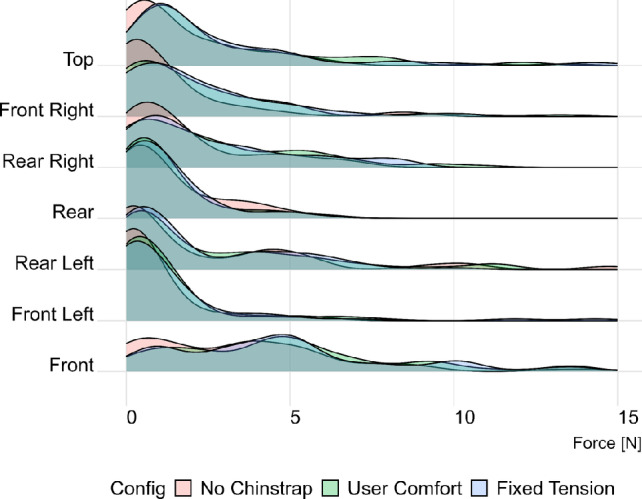
Probability density function of contact force on each interior helmet pad for different wearing configurations

The distribution of contact forces on each pad in the helmet for the three different test configurations is shown in Fig. 2. The distribution for all pads is highly skewed, with the majority of values between 0 and 10 N, and a limited number of higher outliers. Across most pads, a rightward shift can be observed between the no-chinstrap configuration and the configurations with the chinstrap tightened. Additionally, for the user-comfort configuration, the average chinstrap tension measured was 3.4 ± 2.1 N, highlighting a wide range in the users’ preferred helmet tightness.

**Fig. 3:**
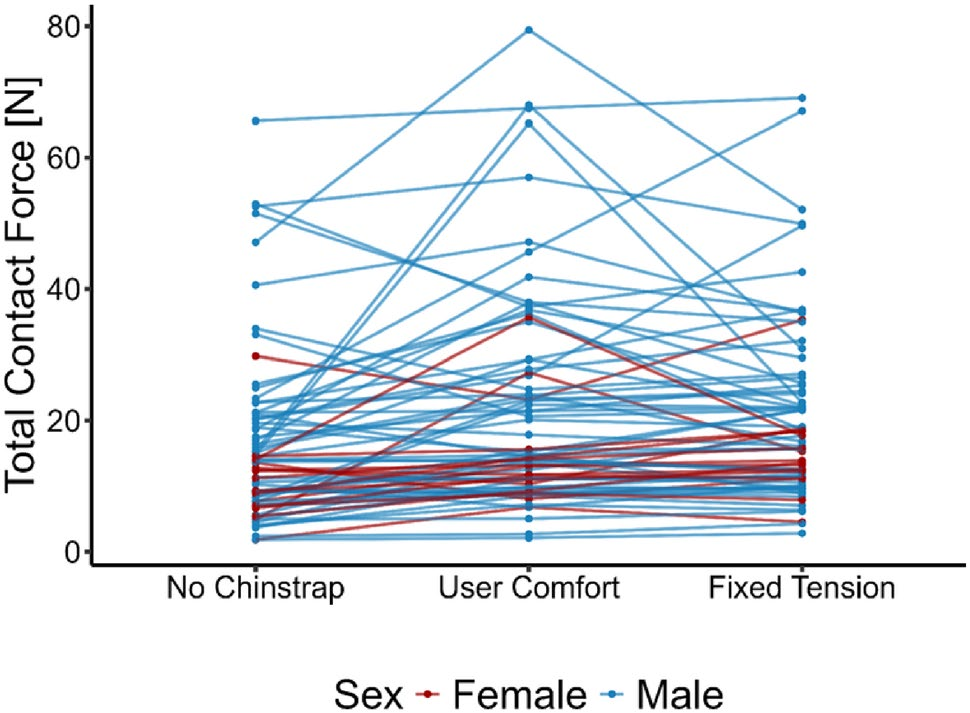
Total contact force versus test configuration, tracking subjects

Figure 3 shows the contact forces for every volunteer in the three different configurations measured. For each volunteer, the average contact force was calculated by summing the force across all pads and dividing by the number of pads. Each line in the plot represents the average force measurements across all pads of a single volunteer for each test configuration. As seen in Fig. 3, the normalized contact force for most volunteers remains consistent. Contact forces between configurations for each subject vary by an average of 24%, compared with a 56−62% variation between subjects. Individuals with contact forces in the lower two quartiles show a minimal change in contact force across different wearing configurations. In the upper quartiles, a larger contact force change between configurations can be observed for the same individual but the variations observed are still smaller than the variation subject-to-subject.

**Fig. 4:**
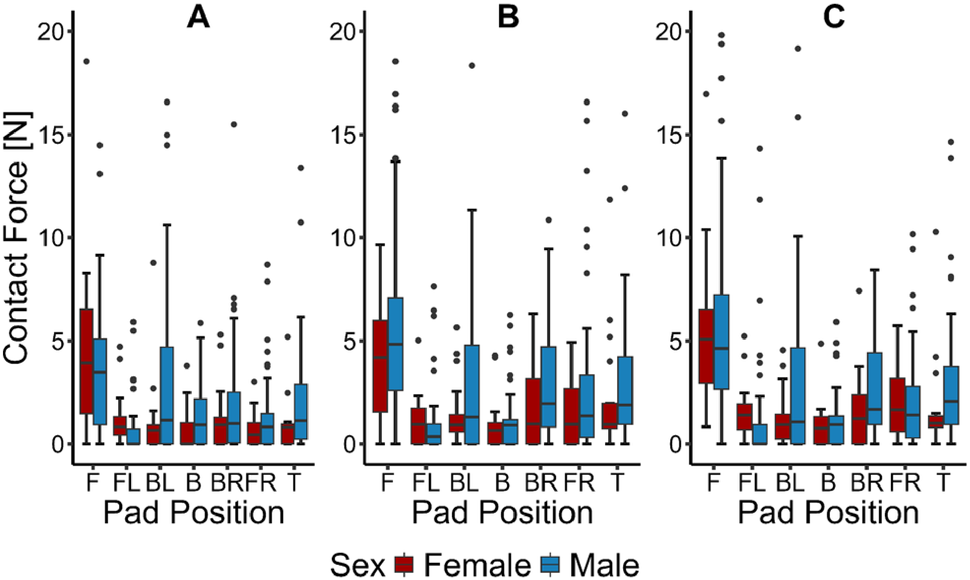
Contact force distributions for each pad for **A** no-chinstrap **B** user-comfort and **C** fixed-tension conditions. F=Front, FL=Front Left, BL=Back Left, B=Back, BR=Back Right, FR=Front Right, T=Top

**Fig. 5:**
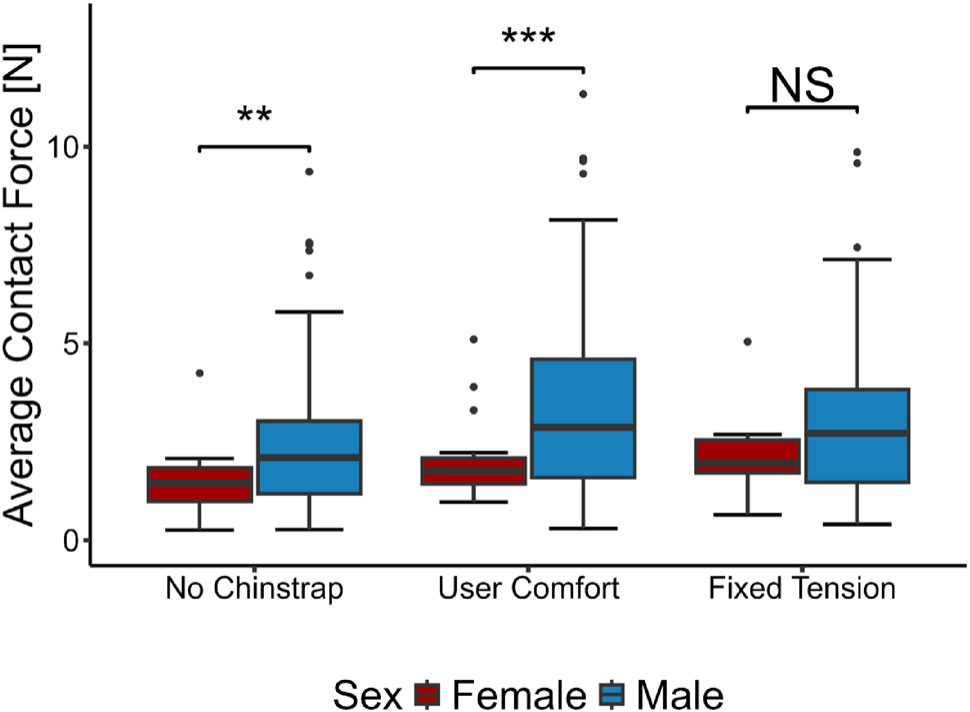
Comparison of average contact force between male and female volunteers for different configurations (no-chinstrap: $p=0.001$, user-comfort: $p=0.0003$, fixed-tension: $p=0.146$).

The distributions of contact forces for each pad in each configuration are shown in Fig. 4. The contact force is highest on the front pad, with the top, back right, and back left having approximately equivalent distributions. There are only minor changes in each distribution between each configuration, consistent with the results shown in Fig. 3. There is a significant spread in the data with a large number of high outliers. Figure 3 and Fig. 5 show that when the contact force is averaged across all seven pads, many of the outliers disappear. Higher-than-normal contact forces in one area of the helmet are compensated by a lower-than-normal contact force elsewhere, indicating that the observed spread in the data is heavily dependent on head shape.

**Fig. 6:**
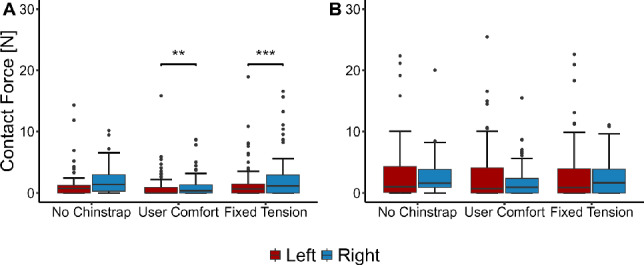
Comparison of **A** front left and right contact forces and **B** rear left and right contact forces for each wearing configuration. Statistically significant differences were observed between front left and front right for the user-comfort ($p=0.005$) and fixed-tension ($p=0.0005$) configurations .

**Fig. 7:**
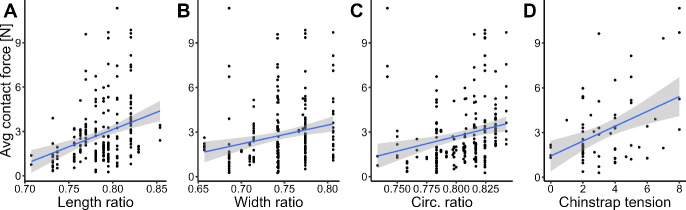
Average contact force versus volunteer **A** head length ratio, $R^2=0.101$ **B** head width ratio, $R^2=0.049$ **C** head circumference ratio, $R^2=0.051$ **D** chinstrap tension, $R^2=0.034$.

A comparison between the left and right pads is shown in Fig. 6. A statistically significant difference between the front left and front right forces was observed for the user-comfort and fixed-tension configuration. No statistically significant different was observed left-to-right in the rear of the helmet. To assess the relationship between head size and the contact forces observed, three normalized dimensions were defined. The length ratio was defined by dividing the front-to-back length of the helmet by the length of the user’s head. Similar definitions were used for the width and circumference ratios. The average contact force across all pads is plotted against these three normalized dimensions in Fig. 7. Linear regression was performed to assess the relationship between each dimension and the contact forces. As each ratio increases, meaning that the head is occupying more of the internal volume of the helmet, the observed contact forces tend to increase. However, the largest correlation coefficient of the three ratios is 0.101, which indicates a weak relationship between the head dimensions measured and the resultant contact forces.

The average contact forces of male versus female participants are compared in Fig. 5. Due to the skewed distribution of the data, non-parametric methods were primarily used for statistical testing. Differences between male and female participants for each configuration were compared using the Mann-Whitney *U*-test [37]. There was a statistically significant difference between male and female volunteers for the no-chinstrap and user-comfort configurations, but no significant difference between sexes for the fixed-tension configuration.

**Fig. 8:**
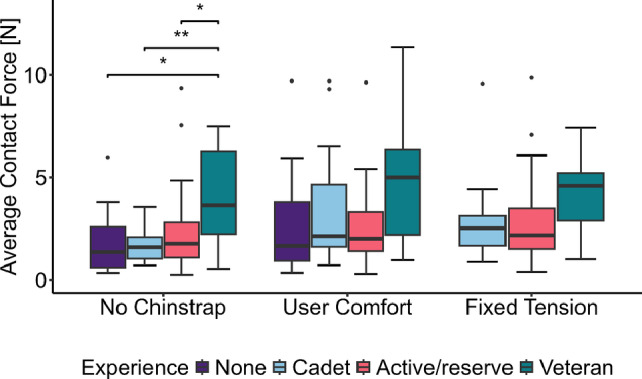
Comparison of average contact force between experience levels for different configurations (no-chinstrap: $p=0.031$, user-comfort: $p=0.137$, fixed-tension: $p=0.061$).

Contact forces were also stratified by the users’ level of experience, as shown in Fig. 8. As discussed above, fixed-tension data were not collected for volunteers without prior experience. Kruskal-Wallis tests [38], a non-parametric alternative to one-way ANOVA [39], were performed comparing different experience levels for each configuration. If an overall statistical difference was found, post-hoc testing was done to determine pair-wise differences. Significant differences were observed for the no-chinstrap configuration between the veteran volunteers and all three other groups. Additionally, no statistically significant difference was observed between groups for the user-comfort or fixed-tension configurations.

## Discussion

The skewed contact pressure distribution observed in the collected data is consistent with expectations, since there is a finite lower bound on the possible contact force but no upper bound. A rightward shift can be observed in most of the pad force distributions shown in Fig. 2 between the no-chinstrap and the user-comfort and fixed-tension configurations indicating that fastening the chinstrap tends to increase the contact forces, consistent with physical intuition. However, the chinstrap configuration does not entirely describe the distribution observed in the data. As shown in Fig. 3, when the total contact force across all pads is averaged, we observed that volunteers with high contact force in one configuration tend to have similarly high contact forces in other configurations. The same is true for individuals with low contact forces. Such a result suggests that the tension of the chinstrap is not a significant driver of the contact force behavior observed in the helmet shell, as confirmed by Fig. 7. Rather, it is likely that most of the subject-specific variation in contact forces observed are due to differences in head geometry.

As seen in Fig. 4, the front pad of the helmet has a much higher contact force than any of the other pads in the helmet. The highest forces tend to be located on the front pad and the back left and right pads, suggesting that the helmet is supported by a triangle of points on the head rather than an even distribution. Different helmet liner kits for the type of Kevlar composite helmet used feature different padding arrangements than the 7-pad distribution shown in Fig. 1. It is possible that different padding arrangements may result in a different triangle of support points or other polygonal patterns. On the other hand, the distribution observed is consistent with previous results collected by Jadischke [29], who observed a similar variation in contact pressure data in football helmets. Notably, the spatial distribution of contact forces is also similar between the data we have collected and the work of Jadischke, with high contact force on the front of the head and the back left and right and low forces on the crown. Since a similar load distribution has been observed in a helmet system with a significantly different padding geometry, it is likely that the distribution observed is, at least in part, due to differences in shape between the head and the helmet shell itself.

We observed statistically different contact forces between the front left and front right pads, as shown in Fig. 6. The left-right differences were consistent across helmet sizes, ruling out equipment malfunction or pad asymmetry as a possible cause. Given that the statistically significant difference is present only when the chinstrap is on it is possible that the chinstrap, which was asymmetric with a buckle on only one side of the head, somehow biased the contact forces on the front of the helmet. However, the left-to-right difference in chinstrap tension in the user-comfort configuration is small: 3.45 ± 2.13 N for the left front strap and 3.33 ± 1.98 N for the right front strap, respectively. Given that the left and right chinstrap tensions were very similar in both cases, further research is necessary to define why such a difference has been observed.

We found no correlation between the observed contact force and the chinstrap tension for the user-comfort configuration, indicating that the chinstrap tension does not significantly affect the observed contact forces. Similarly, there was no correlation between contact force and the length, width, or circumference of the volunteers’ heads. The lack of correlation between any of the metrics we assessed and the contact forces indicates there are one or more additional factors we did not evaluate which affect the contact force. The head dimensions measured—length, width, and circumference—were selected as these three measurements are used to select the correct size helmet for a specific user per the operator manual [36]. The three measurements taken do not fully define the shape of a user’s head, but are still used to select a helmet size. It is clear from Fig. 7 that such dimensions do not correlate well with the true fit of the helmet. By measuring the length, width, and circumference of the volunteers’ heads, we have effectively described their head shape by an ellipse in a coronal plane passing through the glabella and the farthest extreme of the back of the head. As such, we did not capture measurements of head shape in the axial or sagittal planes. It is possible that such measurements may be better predictors of the helmet contact forces by capturing the shape of the region of the head fully inside of the helmet. Previous research to redefine the sizing criteria used has still focused on the use of head length, width, and circumference to define the appropriate helmet size [30]. However, there may be other head measurements which are closely correlated with the observed head-to-helmet forces. Research into relationship between other head measurements and helmet fit may yield sizing criteria which produce a more consistent fit between head and helmet.

The contact forces were significantly higher for male participants compared with female participants for the no-chinstrap and user-comfort configurations, shown in Fig. 5. Such a result could be due to sex-specific differences in head shape, due to the female volunteers having longer hair, or due to differences in preference. The lack of a significant difference between male and female participants for the fixed-tension configuration, where the helmet chinstrap was adjusted by the researcher, suggests that the differences observed in the no-chinstrap and user-comfort configuration are at least partially due to a sex-correlated preference. Because the facilitation instructions focused on measuring the forces under the volunteers’ preferred conditions, the measured results indicate that there are comfort differences between male and female users.

It is possible that some of the statistical differences observed may be affected by the smaller sample size of female volunteers compared with the male volunteers. Only 28% of volunteers were female, and that proportion drops to 22% when considering only volunteers with prior experience using composite helmet systems. While we were not able to achieve a 1-to-1 equal sex representation, our volunteer population provides a higher representation than the proportion of females in roles which may employ this type of helmet [40, 41].

We observed statistically significant differences between veteran volunteers and all other groups. The higher forces observed in the veteran group may be due to greater experience wearing the helmet system leading to a desire for a more snug fit. We did not find significant differences by experience level in the user-comfort and fixed-tension configurations, as seen in Fig. 8. The lack of significant differences suggests that experience level is not a major contributor to the population variance seen in Fig. 3. However, our experience level groupings are not necessarily precise, and a more precise quantification of experience could yield more specific results. Overall, it appears that the average contact force is affected by different and potentially more precise variables.

We have assessed the head/helmet contact force for one type of composite helmet and helmet padding. The composite Kevlar helmet shell assessed shares the same geometry as standard issue helmets used by military and law enforcement. However, differences in padding placement and selection of padding type will result in different contact forces. Particularly, it is likely that changes in thickness from different padding models would change the spatial distribution of contact forces. Kuo et al. found that reducing the padding thickness led to a larger decrease in front and back contact force compared with side or top forces due to less anterior–posterior compression [34]. In addition to the liner system, the shape and weight of the helmet shell likely play a role in determining the contact forces.

Our study focused on the relationship between helmet fit and the forces at the head/helmet interface. As discussed in the introduction, details of the geometry and the relative shape of the head and helmet are also necessary to fully define the quality of helmet fit. Future work to map the relationship between the geometry and the forces observed may yield better fit criteria. Additionally, in future research, the relationship between the helmet fit and the risk of brain injury for different types of impacts should be considered.

## Conclusions

In the present study we—for the first time—provided a quantitative description of the distribution of head/helmet contact forces in a Kevlar composite helmet system with experience level and biological sex. The salient takeaways from our study are as follows:

- The distribution of contact forces was non-uniform across the helmet padding with large forces at the front, back left, and back right.
- The observed contact forces were largely invariant of the configuration with which the helmet was worn, indicating that the head-to-helmet contact force is a function of head shape.
- We observed significant differences between the forces on the front left and front right pads.
- Statistically significantly higher contact forces were observed in male volunteers compared with female volunteers for the no-chinstrap and user-comfort configuration.
- Statistically significant differences were observed between volunteers who were veterans and other volunteers for the no-chinstrap configuration.
- We observed no significant correlation between any of the head measurements taken and the resultant contact forces. Similarly, there was no correlation between the chinstrap tension measured and the contact forces measured.

Our results highlight the importance of considering population variation when evaluating the protective performance of helmets used by military and law enforcement. Additionally, the lack of correlation between the helmet fit and our collected dimensions of the volunteers’ head emphasizes the need for a review of the existing helmet sizing criteria. Our study provides the basis for future investigation into the relationship between helmet ergonomics and protective performance.

## Supplementary Information

Below is the link to the electronic supplementary material.

Supplementary file 1 (pdf 459 KB)

## Data Availability

The raw contact force data analyzed in the present study are available at 10.6084/m9.figshare.24649272.

## Appendix A Educational Materials: Statistical Analysis of Helmet Fit Data

In our recent publications, we have included educational components to broaden the impact of our research [42–48]. To extend such an effort, the following “homework problem" aims to help students practice their skills in statistical analysis using real-world datasets. This assignment is designed for an introductory undergraduate-level course in statistics, measurement systems, or similar experimentalist courses:

The statistical significance shown in Fig. 5 was calculated using the Mann–Whitney *U*-Test [37], an alternative to the *t* test which does not require an assumption that the data are normally distributed. Using the raw data provided in the data availability section, recreate Fig. 5. Using an unpaired t test, evaluate if there is a significant difference between male and female helmet users for each helmet configuration. Are the p values calculated while assuming a normal distribution different from those shown in Fig. 5? Based on the distributions shown in Fig. 2, explain why your results may be different than the results of the Mann-Whitney *U*-tests performed in the manuscript.

## Citation Diversity Statement

Recent research across various scientific fields has revealed a bias in citation practices, showing that papers authored by women and other minority scholars are cited less frequently relative to the number of such papers in the field [49–56]. In our study, we aimed to select references that represent the diversity of the field in terms of gender and race/ethnicity. First, we determined the predicted gender of the first and last authors of each reference using databases that provide the likelihood of a first name being associated with a woman [53, 57]. According to this measure (excluding self-citations to the first and last authors of our current paper), our references include 14.13% woman(first)/woman(last), 14.79% man/woman, 14.6% woman/man, and 56.48% man/man. This method has limitations: a) the names, pronouns, and social media profiles used to create the databases may not always accurately indicate gender identity, and b) it does not account for intersex, non-binary, or transgender individuals. Second, we determined the predicted racial/ethnic category of the first and last authors of each reference using databases that provide the likelihood of a first and last name being associated with an author of color [58, 59]. According to this measure (excluding self-citations), our references comprise 14.49% author of color (first)/author of color(last), 14.92% white author/author of color, 16.65% author of color/white author, and 53.94% white author/white author. This method has limitations: a) names and Florida Voter Data used for predictions may not always accurately indicate racial/ethnic identity, and b) it does not account for indigenous and mixed-race authors or those who might face biases due to ambiguous racial or ethnic names. We anticipate future research will enhance our understanding of how to support equitable practices in science.
