# Supplementary material for: Measurement and Assessment of Head-to-Helmet Contact Forces
Source: Ann Biomed Eng. 2025 Jan 25;53(4):946–55. doi: 10.1007/s10439-025-03677-3 (PMC11929699; doi:10.1007/s10439-025-03677-3)
Supplement: Supplementary file 1 — (pdf 459 KB) [file 10439_2025_3677_MOESM1_ESM.pdf]

Head-to-Helmet Contact Force Measurement in Helmets

Supplementary Material: Helmet FSR Calibration

Equipment setup

Each of the four helmets (size S, M, L, and XL) were fitted with seven foam liner pads (Oregon Aero, Inc., Scappoose, OR). Each liner system consisted of pads with three different geometries as shown in Figure 1: one circular pad at the crown of the helmet, two trapezoidal pads at the front and back, and four oval pads at the front left, front right, back left, and back right. Each pad was instrumented with a force sensitive resistor (FSR) (Adafruit Industries, LLC. New York, NY) by attaching an electrical tape flange to the sides of the FSR and sewing the flange to the fabric covering of the pad as shown in Figure 1. Such a setup fixed the FSR position while allowing it to flex and conform to the surface of the compliant foam. Measurement of the FSR output was facilitated by an Arduino MEGA 2560.

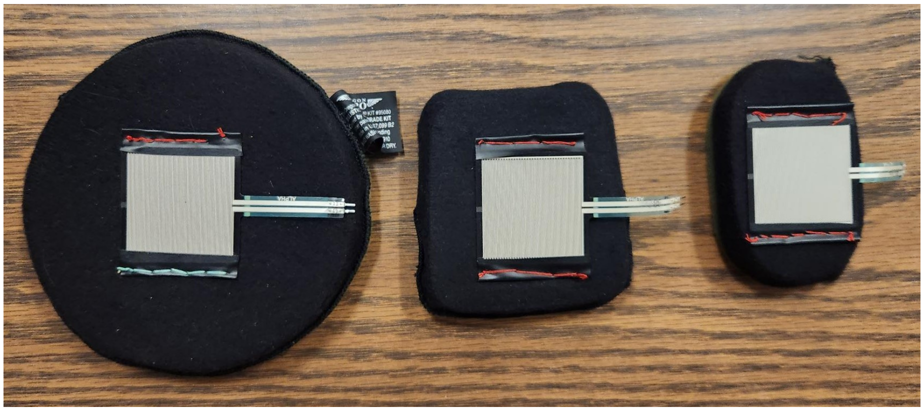

Figure 1: Oregon Aero foam liner pads instrumented with contact pressure sensors. Left to right: Circular top pad, Trapezoidal front/back pad, oval side pad

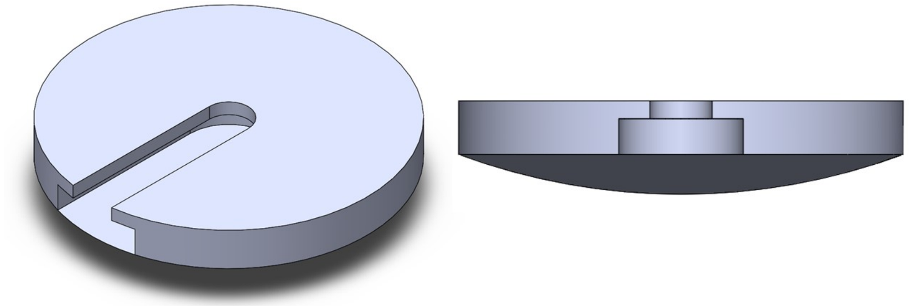

Figure 2: Force applicator used to emulate contact with the curved surface of the head

Calibration Method

A digital force gauge (Imada, Inc., Northbrook, IL) was used to measure the force applied to the FSR. A curved force applicator shown in Figure 2 was fitted to the force gauge to emulate the curvature of the head. The force gauge was mounted on a caliper stand (Mitutoyo America Corp., Holyoke, MA) which was used to apply variable compression to the padding. For each compression level prescribed using the caliper stand, the applied load was measured using the force gauge and the resultant FSR reading was measured using the Arduino. For each of the four helmets, the calibration curves for one of each pad geometry was derived.

Calibration Results

The calibration curves across all helmet sizes and pad geometries showed a similar behavior, so a single calibration curve was derived for all pads using the lumped data from all the pads tested. A cubic spline was found to have the lowest RMS error when fitting the data so was used to relate the FSR analog value to sensor force.

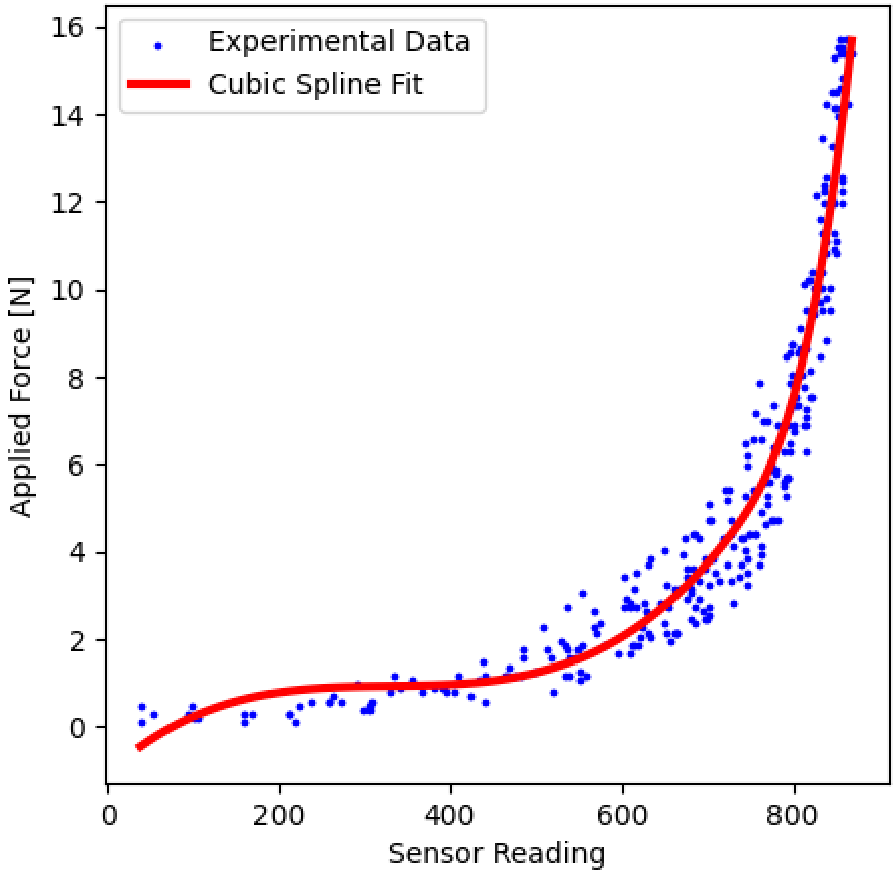

Figure 3: Calibration curve for helmet FSRs
